# Chemoradiotherapy completion and neutropenia risk in HIV patients with cervical cancer

**DOI:** 10.1097/MD.0000000000011592

**Published:** 2018-07-27

**Authors:** Ines Vendrell, Arlindo R. Ferreira, André N. Abrunhosa-Branquinho, Patrícia Miguel Semedo, Catarina F. Pulido, Marília Jorge, Maria Filomena de Pina, Conceição Pinto, Luís Costa

**Affiliations:** a Hospital de Santa Maria, Centro Hospitalar Lisboa Norte; b Luis Costa Lab, Instituto de Medicina Molecular, Faculdade de Medicina, Universidade de Lisboa; c Hospital da Luz, Lisboa, Portugal.

**Keywords:** AIDS, cervical cancer, chemoradiotherapy, HIV, neutropenia risk, oncology

## Abstract

Cervical cancer (CC) is one of the acquired immunodeficiency syndrome (AIDS) defining diseases and the human immunodeficiency virus (HIV) infection is thought to relate with increased acute toxicity of chemoradiotherapy (CRT).

We investigated the effect of HIV status in the incidence of neutropenia associated with cisplatin-based CRT for CC and its impact in treatment completion.

This is a single-center retrospective cohort study. Data collection was performed for all the consecutive stage Ib-IV CC women treated with cisplatin-based CRT from 2012 to 2016, and with known HIV status.

Sixty-one patients were included, 6 were HIV+. HIV+ patients had a higher risk of neutropenia at any cycle during cisplatin CRT [adjusted odds ratio (OR) 7.3, 95% confidence interval (95% CI) 1.02–52.3; *P* = .05]. Despite the absolute differences, mean neutrophil count was nonsignificantly lower in HIV+ women, both at baseline [4455/μL (interquartile range, IQR: 1830–6689) vs 6340 (IQR: 1720–18,970) for HIV−, *P* = .98] and at the end of treatment [1752/μL (IQR: 1100–2930) vs 3147/μL (IQR: 920–18,390) in HIV−; *P* = .06]. Moreover, when considering the effect of time, CRT seems to induce a consistent drop of neutrophils in both groups (*P* = .229). No febrile neutropenia events occurred.

In HIV+ women, there were more CT cycle delays (*P* = .013), patients were more prone to use granulocyte colony-stimulating factor (G-CSF; HIV+ 40.0% vs HIV− 4.0%; *P* = .04) and less likely to complete at least 5 cycles of cisplatin (*P* = .02). All patients received adequate dose of pelvic RT, regardless of HIV status.

HIV+ patients have a significantly increased risk of neutropenia during CRT treatment for CC and are less likely to complete chemotherapy with cisplatin.

## 1 Introduction

Cervical cancer (CC) is one of the most common malignancies in women with human immunodeficiency virus infection (HIV) and its incidence has not decreased with the introduction of highly active antiretroviral therapy (ART) in contrast with Kaposi sarcoma or non-Hodgkin lymphoma.^[1,2]^ Women with HIV have a higher prevalence of infection by human papilloma virus (HPV) and a higher tendency to develop persistent infections.^[3]^ In fact, CC is considered an acquired immunodeficiency syndrome (AIDS) defining disease, and all HIV-positive (HIV+) women with CC have AIDS, regardless of other characteristics.

HIV infection is associated with myelosuppression, and neutropenia occurs in up to 30% to 83% of the patients.^[4,5]^ Pancytopenia caused by HIV infection is multifactorial, partly due to the effect of the infection and replication of the virus in the myeloid progenitor cells and the cytotoxic effect of HIV proteins.^[5]^ Also, the deregulation of the bone marrow microenvironment and of the production of growth factors [with lower circulatory levels of granulocyte colony-stimulating factor (G-CSF)] are potential causes of cytopenia.^[5]^ Moreover, both neutrophils and monocytes might present altered functions.^[6]^

Neutropenia correlates with the degree of immunosuppression^[7]^ and constitutes a risk factor for secondary infections. Hospitalization secondary to a bacterial infection is significantly higher for neutrophil counts under 750 cells/μL [grade 3–4 neutropenia, using common terminology criteria for adverse events version 4.0 (CTCAE v4.0)].^[5]^ Simultaneously, cancer treatment is a competing cause of neutropenia. Locally advanced CC patients undergo concomitant chemoradiotherapy (CRT) and this combination is more toxic than any of the isolated treatments.^[8]^ There are predefined factors that increase neutropenia risk, such as age, comorbidities, previous chemotherapy (CT), active immunosuppression, creatinine clearance, aspartate aminotransferase (AST), and pre-CT bilirubin levels.^[9]^

HIV infection might potentiate CRT-induced neutropenia, which in turn can influence the course of therapy, the risk of infection, and hospitalization. This is especially important, as studies suggest that CC patients receiving fewer than 5 cycles of cisplatin have a decreased overall survival (OS) when compared with those who completed the treatment.^[10,11]^

A previous study concluded that HIV+ patients are less likely to receive guideline-directed treatment for gynecological malignancies,^[12]^ while another study suggested that HIV+ patients with CC were more likely to receive radiotherapy (RT) alone and to develop at least 1 grade 3 to 4 CTCAE v4.0 toxicity during RT or CRT.^[13]^ HIV+ patients were also more prone to stop treatment before its completion. However, this study included patients treated with RT only or undergoing CRT with carboplatin and this agent is known to have higher myelotoxicity than cisplatin.^[14]^ Nevertheless, studies in rectal, anal cancer^[15–17]^ and lymphoma^[18]^ have conflicting results, with some reporting similar rates of toxicity in HIV+ patients, while others report increased rates, especially of myelotoxicity. Recently, the National Comprehensive Cancer Network (NCCN) published guidelines regarding treatment of cancer in people living with HIV,^[19]^ which state that CC should be treated according to the same guidelines as patients without HIV.

If the risk of neutropenia is in fact higher in HIV+ patients, this might influence CT completion, which seems to impact OS. A detailed knowledge about the pattern of neutropenia incidence and its impact on CRT completion can influence the use of hematological support during treatment, which might reflect on treatment outcomes.

To our knowledge, the impact of HIV infection in neutropenia induced by cisplatin-based CRT in CC is not well characterized, which limits informed decision making and the design of treatment guidelines and clinical trials. Therefore, in this study, we aimed to evaluate the impact of HIV infection in neutropenia development during CC treatment with cisplatin-based CRT. We also analyzed the incidence of febrile neutropenia and hospitalization due to infection, CT and RT completion and treatment delay due to neutropenia.

## 2 Methods

### 2.1 Study design/data source

This is a single-center retrospective cohort study. Data concerning patients treated at Centro Hospitalar Lisboa Norte (CHLN) were retrieved. CHLN is not only a reference-center for patients of the Lisbon metropolitan area (referred from primary care and the gynecological oncology multidisciplinary meeting) but also for patients from Portuguese-Speaking African Countries (PALOP), who are medically referred for cancer treatments not available locally.

### 2.2 Patient selection/population

Consecutive women with age 18 years or older and a new histological diagnosis of CC stage Ib-IV (by computer tomography and magnetic resonance imaging) treated with RT at the radiation department and evaluated at the oncology department of CHLN between January 2012 and December 2016 were included. Exclusion criteria included absence of HIV testing and not having undergone cisplatin-based CRT. HIV testing is routinely recommended in Portugal for women with CC, as this is an AIDS-defining disease. Nevertheless, information on HIV testing was not available for all patients, either reflecting absence of testing or screening in another medical facility. Women with small cell CC were also excluded.

### 2.3 Study outcomes

Primary outcomes were any neutropenia event, mean absolute neutrophil count variation, CT completion, and having received adequate whole pelvic radiotherapy (WPRT). Secondary outcomes included treatment delay and hospitalization due to infection, incidence of febrile neutropenia, and total radiation dose in conventional fractionation equivalent dose (EQD_2_)^[13]^ assuming α/β = 10 Gy for the tumor. Total EQD_2_ included all RT delivered in each patient including brachytherapy (BT).

Neutropenia was defined according to the CTCAE v4.0, as neutrophil count under 2000/mm^3^. CT completion was defined as having received 5 of more pre-planned cycles of cisplatin. Adequate WPRT was defined as receiving at least 45 Gy in 25 fractions.^[20,21]^

#### 2.3.1 Covariates of interest

We collected demographic data, including age, ethnicity, and disease stage at diagnosis [International Federation of Gynecology and Obstetrics (FIGO) 2009 staging system]. We identified patients who had received previous CT or RT. Concomitant intake of immunosuppressants, basal bilirubin, AST, and creatinine clearance were collected. We registered information regarding CT administration, neutrophil count before each cycle, and cycle delay. We registered RT data including dose and fractionation regarding external beam RT (EBRT), BT, or external beam boost. Febrile neutropenia was defined as the presence of fever (≥38.5°C) of unknown origin or with microbiologically documented infection, with neutrophil values < 1000/mm^3^. Regarding HIV+ patients, we also collected data regarding ART during CRT and baseline CD4 T cell count.

### 2.4 Treatment

#### 2.4.1 Chemotherapy

Patients were given cisplatin 40 mg/m^2^ (maximum 70 mg/cycle) intravenously in a 500 mL 0.9% sodium chloride (NS) injection over 60 minutes weekly for 6 doses, during weeks 1 to 6 of RT. One thousand milliliters of 0.9% NS were administered with 2 g of magnesium sulfate and 20 mEq/L of potassium chloride over 90 minutes, before and after cisplatin. Antiemetic prophylaxis was given according to the European guidelines for antiemesis in high emetogenic potential CT.^[22]^ Cisplatin would not be administered if serum creatinine was > 1.5 times the upper limit of normality or if neutrophils were ≤ 1500/mm^3^.

#### 2.4.2 Radiotherapy

RT was delivered as WPRT followed by a boost to the tumor with BT or with EBRT if BT was unfeasible (risk of uterine perforation, anesthesia-associated risks, insufficient tumor response for BT applicator insertion, or patient refusal). In case of hemostatic intention, a first EBRT plan was delivered to the tumor, and a second planning-CT was acquired for WPRT. All patients underwent a pelvic CT-scan for WPRT EBRT with 3 mm image thickness reconstruction. Target volumes were in accordance with International Commission on Radiation Units and Measurements (ICRU) Reports #50 and #62 and all patients were treated with conformal EBRT (3D-CRT). Dose prescription and fractionation selection was according to clinical judgment (45–50.4 Gy in 25–28 fractions).

BT patients were submitted to general anesthesia to insert tandem and other adequate devices. ICRU #38 recommendations were followed. High dose rate BT was delivered with an iridium-192 source. Dose prescription and fractionation varied due to institutional policy along the years (24 Gy/4 fractions or 21 Gy/3 fractions). If BT was unfeasible or considered an incomplete treatment, patients would undergo an additional planning-CT to plan a boost with 3D-CRT technique. Dose prescription and fractionation were dependent of clinical judgment (9–16.2 Gy in 5–9 fractions).

This project was ethically approved, complied with all local regulations, and is in accordance with the 1964 Helsinki declaration and its later amendments. No formal consent was required by the ethics committee, given the nature of the study.

### 2.5 Statistical analysis

Patients were divided into 2 cohorts. Cohort 1 included HIV+ patients and cohort 2 included HIV 1/2 negative (HIV−) patients.

Descriptive statistics of baseline demographic, clinical, pathological, and treatment characteristics were performed, according to HIV status. We tested differences in groups with Fisher exact test or Wilcoxon rank-sum, as appropriate. The variation of neutrophil count was evaluated in each cycle. For dichotomous outcomes, univariate and multivariate analyses [including HIV status, baseline AST, estimated glomerular filtration rate (eGFR), and total EQD_2_ of RT] were performed using logistic regression. Goodness of fit of the models was evaluated by the Hosmer–Lemeshow test and ROC curves. Mixed linear models were used to determine the effect of HIV and time on neutrophil count. Data were analyzed using Stata v14.2 (StataCorp LLC Texas). *P* values less than .05 were considered statistically significant.

## 3 Results

### 3.1 Study sample and baseline characteristics

**Figure 1:**
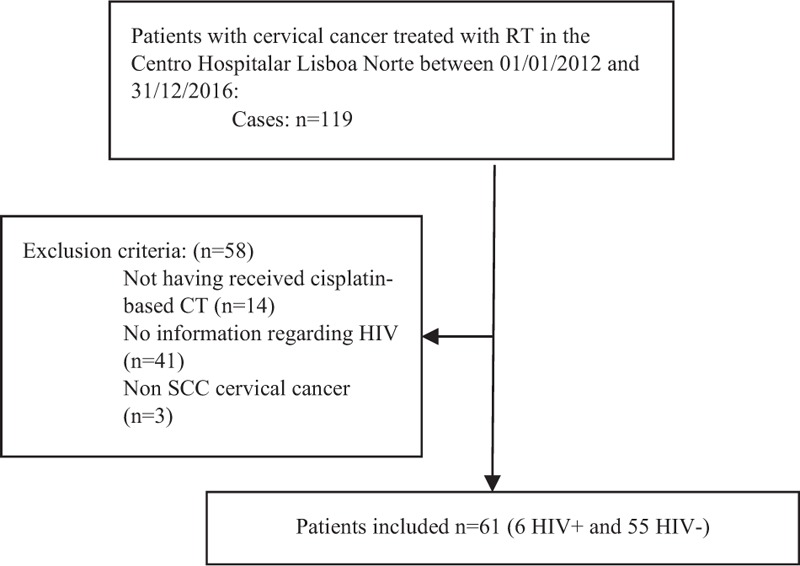
Study design and exclusion criteria (CONSORT).

A total of 119 patients with CC were identified, of whom 58 were excluded; 61 were eligible for the study (Fig. 1).

Baseline demographic, clinicopathological characteristics, and previous treatment received are summarized in Table 1. Among the 61 patients included, 55 were HIV− and 6 were HIV+. Mean age was similar in both groups. Most HIV− women were black (65.4%; n = 36), while black women represented 50.0% of HIV+ women (*P* = .66). Regarding the country of origin, most HIV+ patients were from Portugal (50.0%, n = 3), while most HIV− patients were from Cape Verde (52.7%, n = 29).

**Table 1:**
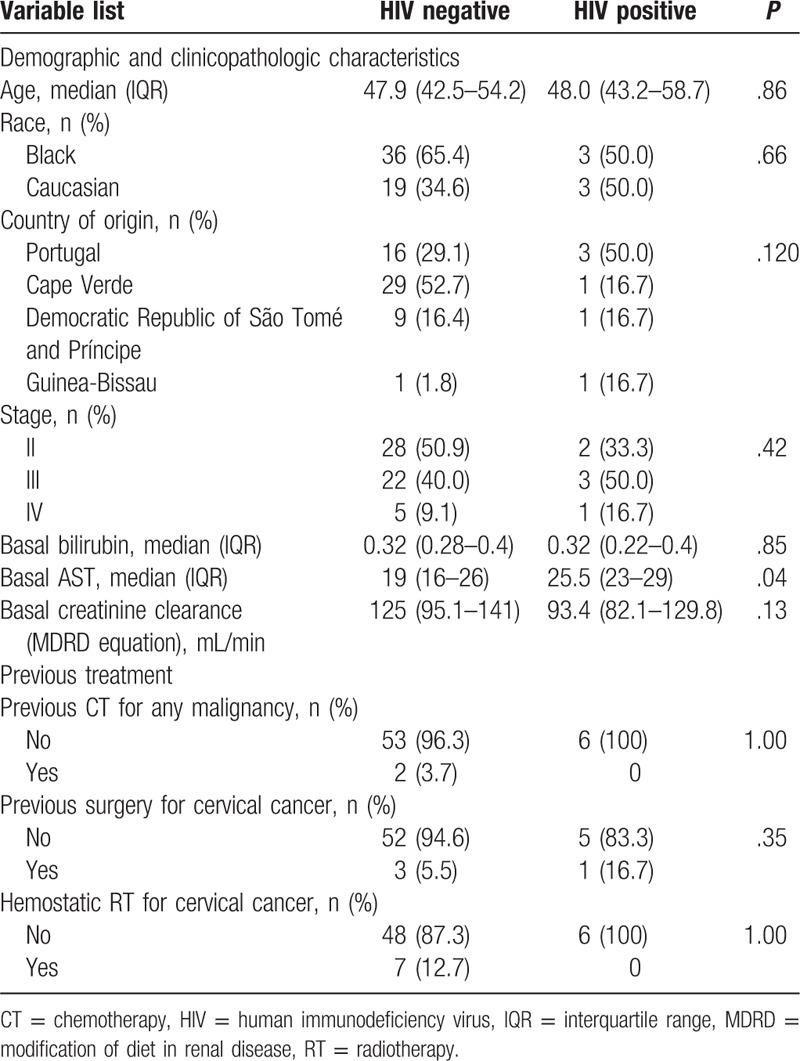
Demographic, clinicopathologic characteristics, and previous treatment.

Regarding clinical stage at diagnosis, the groups were balanced, with most patients classified with stage II-III in both groups (*P* = .42; Table 1).

Most patients had not received previous CT for other malignancy (96.3% HIV− and 100% of HIV+ women). Only 12.7% (n = 7) of HIV− had received previous hemostatic RT for CC, while none of the HIV+ had previous RT. No patient received concomitant immunosuppressant medication during CRT.

Regarding baseline characteristics, there were no differences in univariate analysis between median bilirubin levels (*P* = .85) and eGFR (*P* = .13), calculated through the modification of diet in renal disease (MDRD) formula. AST levels were significantly higher in HIV+ women (*P* = .04), although within the normal range.

### 3.2 HIV+ population

Of the 6 HIV+ patients, 4 were under ART during CRT (2 without ART). The only patient with monotherapy was under a non-nucleoside reverse transcriptase inhibitor (NNTRI). The other 3 were taking combination therapy, 2 under nucleoside reverse transcriptase inhibitors (NTRIs) and protease inhibitors (PIs), and 1 under NTRI and integrase inhibitors (II).

Mean T CD4+ count before CRT was 567.5 cells/mm^3^ (range 59.9–1181.6), and was significantly lower in HIV+ women who were not under ART [mean 69.3 cells/mm^3^, 95% confidence interval (95% CI) -163.9 to 302.4] when compared with women under ART (mean 747.8 cells/mm^3^, 95% CI 271.2–1324.3; *P* = .04). Regarding baseline neutrophil count, HIV+ women without ART had mean 4255/μL, (range 1830–6680/μL) and HIV+ women under ART had mean 4555/μL (range 3560–5700/μL). When analyzing different ART combinations, the patient under NNTRI had baseline 6450 cells/mm^3^, the patient under NTRI and II had 8610 cells/mm^3^, and mean count of patients with NTRI and PI inhibitors (n = 2) was 6185 cells/mm^3^.

### 3.3 Neutropenia

Despite the absolute differences, baseline neutrophil count was nonsignificantly lower at baseline in HIV+ women (mean 4455/μL, range 1830–6689 vs mean 6340, range 1720–18,970 in HIV−; *P* = .86), Table 2.

**Table 2:**
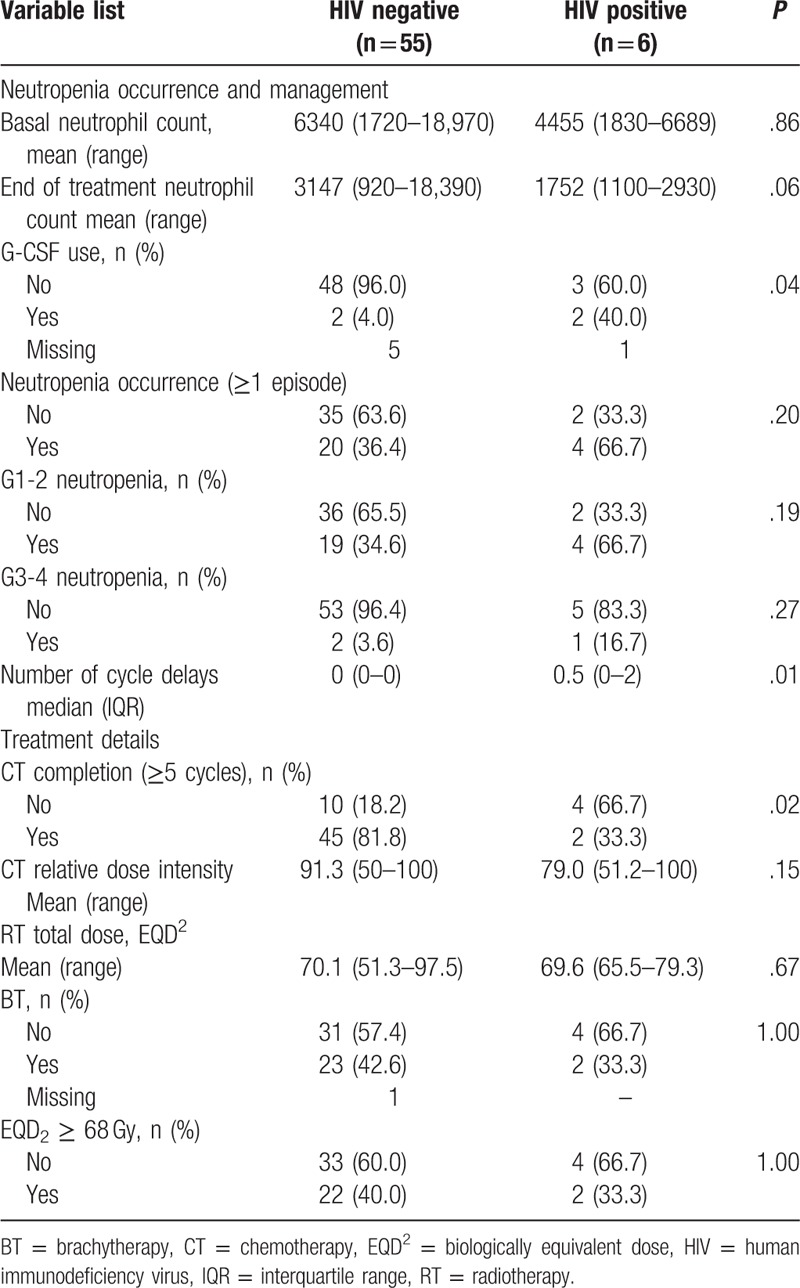
Neutropenia occurrence and treatment details.

**Figure 2:**
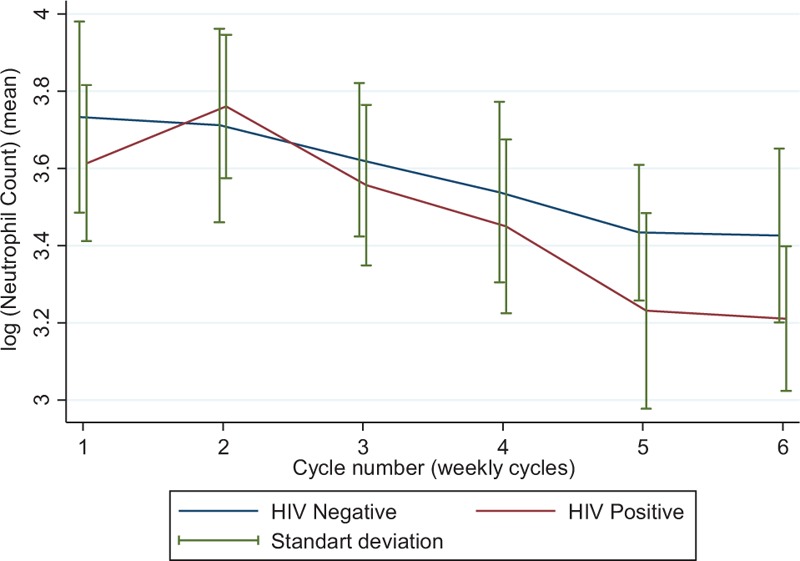
Variation of the mean log (neutrophil) value between cycles according to HIV status.

From cycles 1 to 6, neutrophil count decreased in both groups (*P* < .001; Fig. 2), with neutropenia occurring in 66.7% of HIV+ patients and 36.4% of HIV− patients (univariate *P* = .20). In the multivariate analyses, HIV+ patients had a higher risk for the occurrence of neutropenia [adjusted odds ratio (OR) 7.3, 95% CI 1.02–52.3; *P* = .05]. Model fit was appropriate [area under the curve (AUC) = 0.75].

**Figure 3:**
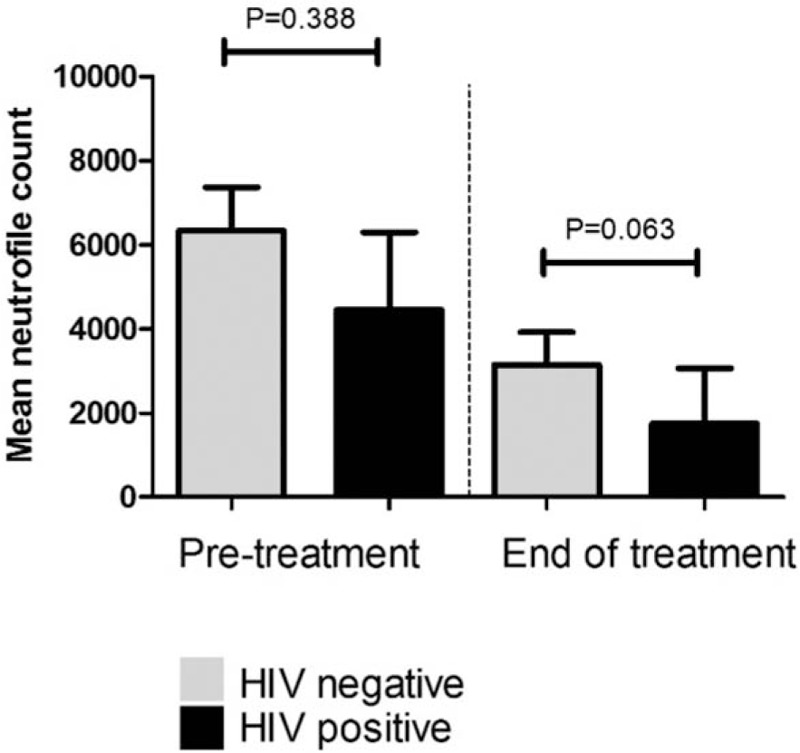
Pre and post treatment neutrophil count according to HIV status.

At the end of treatment, there was a trend for lower median neutrophil values in HIV+ women [1752/μL (IQR 1100–2930) versus 3147/μL (IQR 920–18,390) in HIV− women; *P* = .06], Fig. 3.

Finally, in order to evaluate if the mean neutrophil profiles differed in both groups, we determined the interaction between HIV status and time, but no significant difference was noted (*P* = .229).

### 3.4 Febrile neutropenia and hospitalization due to infection

There were no episodes of febrile neutropenia during CRT treatment, regardless of HIV status.

There was 1 hospitalization due to active pulmonary tuberculosis during CRT in an HIV+ woman who was not under ART, when compared with none of the HIV− women.

### 3.5 Chemotherapy completion

Overall, 66.7% (n = 4) of the HIV+ patients received 5 or more cycles of cisplatin when compared with 81.8% (n = 45) of the HIV− patients. The lower rate of completion of at least 5 cycles of cisplatin was significant in both the univariate (*P* = .02) and multivariate analyses (OR 0.11, 95% CI 0.02–0.76; *P* = .02). There was no evidence of poor fit of the model (*P* = .28), with an AUC = 0.66.

Moreover, HIV+ women had more CT cycle delays (median 0.5, IQR 0–2, *P* = .01) and were also more prone to use G-CSF during CRT (40.0% vs 4.0% in HIV−; *P* = .04). Mean relative dose intensity of CT was nonsignificantly lower in HIV+ patients (79.0%, range 51.2–100.0% vs mean 91.3%, range 50.0–100.0% in HIV− women; *P* = .15) (Table 2).

### 3.6 Radiotherapy completion

Mean total dose of external radiotherapy was 70.1 Gy EQD_2_ (range 51.3–97.5) in HIV− women when compared with 69.6 Gy EQD_2_ (range 65.5–79.3) in HIV+ women (*P* = .67). In both groups, the majority of patients failed to receive BT (HIV+: 66.7%, n = 4; HIV−: 57.4%, n = 31, *P* = 1.00), Table 2. When considering minimum WPRT dose, all patients received at least 45 Gy, regardless of HIV status.

Mean duration of WPRT was significantly lower in HIV− patients, 44.7 days (range 31.0–65.0) when compared with 55.2 days (range 36.0–79.0) in HIV+ women (*P* = .03). Two (33.3%) of the HIV+ women had a RT duration of more than 55 days, when compared with 10 (18.2%) of the HIV− patients.

## 4 Discussion

In this cohort of 61 patients, HIV+ women had a higher risk for the occurrence of neutropenia (adjusted OR 7.3, 95% CI 1.02–52.3) and a nonsignificant, but overall consistent trend for decreased neutrophil counts at baseline, during, and at the end of treatment. When considering the interaction between time and HIV, CRT seems to induce a consistent drop of neutrophils in both groups. Finally, this further translated into a lower rate of chemotherapy treatment completion, more treatment delays, and more frequent G-CSF support, but no detrimental impact on the delivery of planned RT.

Our results are in line with those obtained by previous authors, who concluded that more HIV+ patients experienced serious toxicity, including leukopenia,^[13]^ although in that cohort, HIV+ patients had a 30.6% incidence of grade 3 to 4 leukopenia when compared with 10.2% of HIV− patients (*P* = .04); in our study, the incidence was lower, 16.7% (HIV+) versus 3.6% (HIV−), *P* = .27.^[13]^ Nevertheless, unlike our study, that cohort also included patients treated with RT only and treated with carboplatin, which has a higher myelotoxicity rate than cisplatin and this might in part account for the difference in grade 3 to 4 toxicities.^[14]^ Also, in the same cohort study,^[13]^ most HIV+ patients were unaware of their HIV+ status before CC diagnosis and few were on ART before the first oncology evaluation in contrast to our study. This might have had an impact in the immunological status and could relate to such higher rates of toxicity. In fact, median CD4^+^ count of the HIV+ patients in that study was 354 cells/μL (range 33–1249), when compared with mean cell count of 567.45 cells/μL in our study (range: 59.9–1181.6). We should also point out that there was a significantly higher proportion of use of G-CSF in HIV+ patients in our cohort, which might have contributed to a lower rate of neutropenia, when compared with other cohorts.^[13]^

Nevertheless, there were no differences in both febrile neutropenia rate and hospitalization for infection in both groups.

In our cohort, HIV+ patients were less likely to complete 5 cycles of cisplatin CT. However, the rate of patients who received a minimum dose of WPRT was similar for both groups, as well as the mean EQD_2_ dose of RT. This supports other studies showing that HIV+ patients tend to receive adequate RT but are less likely to complete CT,^[13]^ a finding also reported for HIV+ patients with squamous cell carcinoma of the anal canal (SCCA).^[15,17]^ In SCCA, the CT doses are described to be reduced (in 54–66.6% of patients), although sometimes these were pre-planned reductions. Similar doses of RT are given in both HIV+ and HIV− patients.^[15,17]^

Of note, mean WPRT duration was significantly longer in HIV+ patients (55.2 days). Considering that 33.3% of HIV+ patients and 42.6% of HIV− patients additionally received BT, the total duration of treatment was in fact even longer for these patients. It is known that EBRT and BT should be completed in 55 days or less (8 weeks), as higher duration of treatment is associated with worse outcomes both in tumor control and OS.^[23]^ The duration of RT in our cohort might relate with several factors such as noninfectious medical complications, compliance with treatment, and technical/logistical challenges.

When considering HIV+ women only, although mean T CD4+ count at baseline was significantly lower in HIV+ women who were not under ART (n = 2), there was no difference in rates of CT completion for those who were under ART therapy and those without treatment during CRT, but sample size precludes us from deriving conclusions from these results.

Despite all the methodological efforts, this study has limitations. This is a small, single-center retrospective cohort study. Long-term follow-up was not available due to loss of follow-up of patients medically referred from the PALOP, given that in these cases, further care is provided locally. Although all patients under ART showed neutrophil counts within the normal range at baseline, we were not able to control for the potential myelosuppressive effects of different classes of ART drugs. Also, the unbalanced use of G-CSF in most HIV+ patients might have positively impacted neutrophil counts in this group, thus blunting the effect of HIV infection. The use of G-CSF in CRT is still controversial and most recent recommendations on its use derive from studies in chest malignancies.^[24,25]^ Even if neutropenia is reversed, old reports for abdominal RT and extended field CRT in chest malignancies stated a small number patients had severe thrombocytopenia with the use of G-CSF.^[26]^ This led ASCO to recommend against use of CSFs for CRT, but these recommendations are being questioned.^[24,27]^

In summary, we showed that HIV+ patients have a significantly increased risk of neutropenia during CRT for CC, even while receiving G-CSF more frequently. They are also less likely to complete CT, while receiving similar RT dosing. These data support the need of a careful follow-up of HIV+ patients during CRT treatment given their increased risk for toxicity. Further research on the impact of treatment completion on local recurrence and survival are needed.

## Author contributions

**Conceptualization:** Ines Vendrell, André N. Abrunhosa-Branquinho, Catarina F. Pulido, Marília Jorge, Maria Filomena de Pina, Conceição Pinto, Luís Costa.

**Data curation:** Ines Vendrell, Arlindo R. Ferreira, André N. Abrunhosa-Branquinho, Patrícia Miguel Semedo, Catarina F. Pulido.

**Formal analysis:** Ines Vendrell, Arlindo R. Ferreira.

**Methodology:** Ines Vendrell, Arlindo R. Ferreira.

**Supervision:** Ines Vendrell, Arlindo R. Ferreira.

**Validation:** Ines Vendrell.

**Writing – original draft:** Ines Vendrell, Arlindo R. Ferreira, André N. Abrunhosa-Branquinho, Patrícia Miguel Semedo, Catarina F. Pulido, Marília Jorge, Maria Filomena de Pina, Conceição Pinto, Luís Costa.

**Writing – review & editing:** Ines Vendrell, Arlindo R. Ferreira, André N. Abrunhosa-Branquinho, Patrícia Miguel Semedo, Catarina F. Pulido, Marília Jorge, Maria Filomena de Pina, Conceição Pinto, Luís Costa.
